# Development and evaluation of rapid and simple detection of *Klebsiella pneumoniae* using closed dumbbell-mediated isothermal amplification diagnostic assay

**DOI:** 10.3389/fmicb.2024.1435010

**Published:** 2024-08-07

**Authors:** Yanli Zhang, Xuhan Chen, Guifang Ouyang, Jiaping Wang, Yongcheng Sun, Yanli Lai, Ping Zhang, Fei Guo, Shujun Yang, Rui Mao

**Affiliations:** ^1^Department of Hematology, The First Affiliated Hospital of Ningbo University, Ningbo, China; ^2^Ningbo Institute of Life and Health Industry, University of Chinese Academy of Sciences, Ningbo, China; ^3^Department of Laboratory Medicine, The First Affiliated Hospital of Ningbo University, Ningbo, China

**Keywords:** *Klebsiella pneumoniae*, closed dumbbell-mediated isothermal amplification (CDA), sensitivity, specificity, point of care test

## Abstract

**Introduction:**

*Klebsiella pneumoniae* (*K. pneumoniae*) is the most common pathogen causing hospital respiratory tract infection and epidemic. Gold standard procedures of microscopic examination and biochemical identification are widely used in clinical diagnosis with disadvantages of low sensitivity, time-consuming and sophisticated equipment requiring. An efficient, nucleic acid amplification-based sensitive and specific on-site identification of *K. pneumoniae* in clinical is necessary to facilitate clinical medication and disease control.

**Methods:**

We developed a closed dumbbell mediated isothermal amplification (CDA) assay for the rapid and sensitive detection of conserved *rcs*A gene in *K. pneumoniae* by real-time fluorescence monitoring and end-point colorimetric judgement. We designed and selected a pair of inner primers of CDA to detect *K. pneumoniae*. Then outer and loop primers were designed and verified to accelerate CDA reaction to achieve more efficient detection of *K. pneumoniae*.

**Results:**

The results showed the detection limit of CDA assay was 1.2 × 10^−5^ ng/μL (approximately 1 copy of the target gene) within 60 min, which was 100-fold more sensitive than real-time quantitative PCR (qPCR). Several pathogen genomic DNAs (*Staphylococcus aureus, Shigella sonnei, Vibrio parahaemolyticus, Escherichia coli, Candida glabrata, Candida tropicalis, Candida parapsilosis, Candida albicans, Streptococcus agalactiae, Rickettsia, Listeria monocytogenes*, *Pseudomonas aeruginosa, Klebsiella oxytoca*, and *Klebsiella aerogenes*) were used to evaluate the sensitivity and specificity of the established *K. pneumoniae* CDA assay. Total 224 batches of samples from other strains tested were negative and 296 batches of extracted *K. pneumoniae* DNA samples were positive by the developed CDA amplification approach, revealing high specificity and specificity of the diagnostic assay. In addition, the results of real-time fluorescence amplification of the *K. pneumoniae* CDA were in consistent with those of end-point colorimetric results.

**Discussion:**

The established real-time fluorescence and visual CDA assays of *K. pneumoniae* with merits of rapid, sensitive and specificity could be helpful for on-site diagnosis and clinical screening in rural areas.

## 1 Introduction

*Klebsiella pneumoniae* (*K. pneumoniae*), a Gram-negative bacterium, is ubiquitously found on the mucosal surfaces of animals and humans, causing diseases including urinary tract infections, respiratory tract infections, bacteremia, liver abscesses, and pneumonia (Podschun and Ullmann, 1998; Siu et al., 2012; Snitkin et al., 2012; Wang et al., 2020). As a human commensal and conditioned pathogen, *K. pneumoniae* colonizes the gastrointestinal tract, usually causing infections in immunocompromised or hospitalized patients (Martin and Bachman, 2018; Russo and Marr, 2019). Recently, the emergence of *K. pneumoniae* with a high infection rate in hospitals has become a serious health issue (Gorrie et al., 2017; Zhang Y. et al., 2018). Thus, the development of rapid and accurate diagnostic tools for *K. pneumoniae* is essential to guide appropriate medication and therapy.

Conventional methods for *K. pneumoniae* detection, such as microscopic examination, biochemical identification, and serological detection, were complicated, strenuous, and time-consuming (Bachman et al., 2015; Zhang W. et al., 2018; Wang et al., 2019). Molecular biology-based technologies have been widely adopted for the identification of pathogens, with significant improvements in accuracy and sensitivity over the last few decades. Approaches based on the polymerase chain reaction (PCR) were carried out to achieve a phenotypic diagnosis of *K. pneumoniae* (Yu et al., 2018). Typically, the conserved *rpo*B, *gap*A, and 16S–23S internal transcribed spacer genes were selected for the identification of *K. pneumoniae* through PCR amplification (Diancourt et al., 2005). However, the requirements of skilled operators, complex procedures, and sophisticated equipment hindered the established PCR methods (Weng et al., 2023).

Currently, developments in isothermal amplification-based technologies are aiming to offer alternative methods for *K. pneumoniae* PCR detection with the merits of efficiency, sensitivity, and specificity gradually. Recently, a recombinase-aided amplification (RAA) assay was used to detect *K. pneumoniae* in milk samples by adding an expensive enzyme mixture. Loop-mediated isothermal amplification (LAMP)-based *K. pneumoniae* assays were developed by selecting sophisticated four- to six-oligonucleotide primers to amplify eight distinct regions (Liao et al., 2020). Therefore, considering that complex primer design and expensive recombinase might lead to inefficient application, an efficient, simple, and on-site detection assay of *K. pneumoniae* in clinical settings is necessary for direct and accurate treatment.

To overcome the drawbacks of PCR and LAMP methods, a novel nucleic acid detection technology named the closed dumbbell-mediated isothermal amplification (CDA) assay was established (Mao et al., 2022a). The CDA assay was simpler than other molecular diagnostic technologies as it did not require a thermal cycler, an expensive recombinase, or a long primer sequence (Gui et al., 2022). Compared with other isothermal amplification technologies, the establishment of the CDA assay required a concise and simple primer design to obtain a cost-efficient reaction (Mao et al., 2022b). In this study, we successfully established and evaluated real-time fluorescence and on-site visual-based molecular diagnostics of *K. pneumoniae* using the CDA method.

## 2 Materials and methods

### 2.1 Ethics approval statement

Ethical approval, including the waiver of informed consent for the clinical strains and samples collected in the laboratory of the First Affiliated Hospital of Ningbo University, was approved by the Ethics Committee of Clinical Investigation in the First Affiliated Hospital of Ningbo University under approval number 2024-055A-01. The research conformed to the principles of the Declaration of Helsinki. The study presented no more than minimal risk to subjects, and no personal information was obtained.

### 2.2 Reagents and materials

DNA primers were provided by BGI Biological Engineering Technology and Services Co. Ltd. (Shenzhen, China) or Sangon Biotech (Sangon, Shanghai, China). The *Bst* 2.0 WarmStart DNA polymerase and 10× ThermoPol reaction buffer [including 200 mM Tris–HCl, 100 mM KCl, 100 mM (NH_4_)_2_SO_4_, 20 mM MgSO_4_, and 1% Triton X-100] were purchased from New England BioLabs (Ipswich, MA, United States). A DNA molecular weight marker was purchased from Thermo Fisher Scientific (Waltham, MA, United States). Deoxynucleotide triphosphates (dNTPs), DNA purification kits, and DNA extraction kits were purchased from Sangon Biotech (Sangon, Shanghai, China). The commercial *Klebsiella pneumoniae* Real-Time PCR kit was purchased from Shanghai Liferiver Bio-Tech Co. Ltd. (Shanghai, China). Other reagents, unless specified, were obtained from Sigma-Aldrich (St. Louis, MO, United States).

A total of 30 clinical isolates of *K. pneumoniae* were collected from October 2023 to May 2024 by the research team from the laboratory of the First Affiliated Hospital of Ningbo University. Various non-*K. pneumoniae* clinical isolates and strains are listed in Table 1. Standard procedures for clinical isolates of the bacterium were cultured on Columbia blood agar plates in a CO_2_ incubator for 48 h and were preserved for later use (Luginbühl et al., 2017). The *Candida* strains were cultured in the yeast peptone dextrose (YPD) medium (Li et al., 2021), while *Escherichia coli* was cultured in the Luria-Bertani (LB) medium (Jira et al., 2018). The genome DNA from all isolates was extracted using the DNA purification kits and stored at −20°C. The online DNA copy number calculator^1^ was adopted to determine the copy numbers of targets, and the calculation procedures were carried out following the instructions provided by Zong et al. (2012).

**Table 1:** Bacterial strains used in this study.

Species	Strain	Source	Year
*Klebsiella pneumoniae*	BNCC361251	BNCC	2022
*Klebsiella pneumoniae*	KPC2024 (01 ~ 30)	Collected by our research team	2023–2024
*Staphylococcus aureus*	CGMCC 1.6750	CGMCC	2021
*Shigella sonnei*	CVCC 3926	CVCC	2020
*Vibrio parahaemolyticus*	CGMCC 1.1997	CGMCC	2021
*Escherichia coli*	CGMCC 1.12883	CGMCC	2021
*Candida glabrata*	BNCC 337348	BNCC	2021
*Candida tropicalis*	BNCC 186815	BNCC	2021
*Candida parapsilosis*	BNCC 337317	BNCC	2021
*Candida albicans*	CICC 1965	CICC	2021
*Streptococcus agalactiae*	BNCC 185941	BNCC	2022
*Rickettsia raoultii*	RR202201	Preserved in our laboratory	2021
*Listeria monocytogenes*	CVCC 1597	CVCC	2021
*Pseudomonas aeruginosa*	BNCC336458	BNCC	2022
*Klebsiella oxytoca*	BNCC340160	BNCC	2023
*Klebsiella aerogenes*	BNCC364140	BNCC	2023

BNCC, BeNa culture collection; CGMCC, China general microbiological culture collection center; CICC, China center of industrial culture collection; CVCC, China veterinary culture collection center.

### 2.3 Primer design

**Figure 1:**
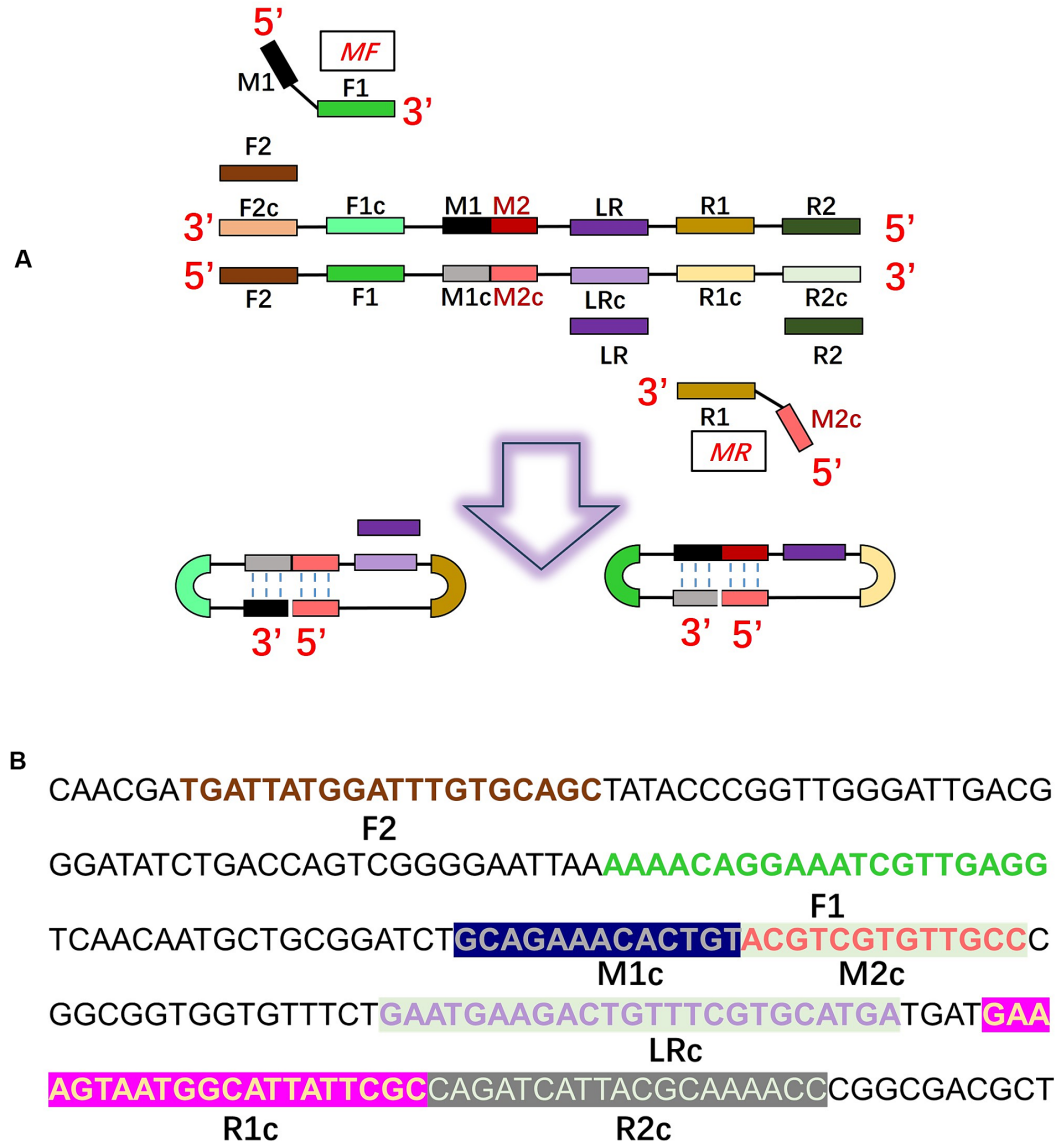
Principle of the closed dumbbell-mediated isothermal amplification (CDA) method for *Klebsiella pneumoniae* detection. **(A)** Overall scheme for primers of *K. pneumoniae* used in the CDA method. **(B)** Partial sequence of the gene of *K. pneumoniae* used for designing the primers of the CDA method.

The CDA primer design procedures are illustrated in Figure 1A. We designed a CDA primer from a sequence of the transcriptional regulator *rcs*A gene of *K. pneumoniae* (GenBank: AY059955.1 and CP131701.1) after alignments (Dong et al., 2015). In the primer design procedure, the sequences of “forward 1,” “reverse 1,” and “middle” were abbreviated as F1, R1, and M, respectively. The site with the lowercase “c” represents “complementary.” The inner sequence between F1c and R1 is considered as M, which is separated into M1 and M2 equivalently. According to this structure, MF comprises a sequence complementary to M1 (M1c) and a sequence complementary to F1c (F1). MR comprises sequences (M2) complementary M2c and R1. Based on this scheme, we selected the optimal primer that was able to amplify the *rcs*A gene in a short time from four designed pairs of primers (Table 2, partially). To promote the CDA reaction, outer primers (F2 and R2) and loop primers (LRs) were designed and selected to optimize the CDA assay. The situations of all selected CDA primers used in this study are shown in different colors in Figure 1B. To compare the sensitivity of the CDA assay and PCR assay, PCR primers were designed with the same template sequences by DNAMAN Version 8.0 software. All sequences of primers are listed in Tables 2, 3, and these sequences are synthesized by Biological Engineering Technology and Services (BGI Biotech Co., Ltd., Shenzhen, China) or Sangon Biotech (Sangon, Shanghai, China).

**Table 2:** Screening of KP-CDA primer sequences for the mapping genes of *Klebsiella pneumoniae*.

Target	Primer	Sequence (5′ → 3′)
*Klebsiella pneumoniae rcs*A gene	KP-MF-1	GTTTCTGCAGATCAAAAACAGGAAATCGTTGAGG
	KP-MR-1	ACTGTACGTCGTGGCGAATAATGCCATTACTTTC
	KP-MF-2	AGAAACACCACCAAAAACAGGAAATCGTTGAGG
	KP-MR-2	GAATGAAGACTGGCGAATAATGCCATTACTTTC
	KP-MF-3	GCATTGTTGACCTCAAAAACAGGAAATCGTTGAGG
	KP-MR-3	TGCGGATCTGCAGGCGAATAATGCCATTACTTTC
	KP-MF-4	ACAGTGTTTCTGCAAAACAGGAAATCGTTGAGG
	KP-MR-4	ACGTCGTGTTGCCCGAATAATGCCATTACTTTC

**Table 3:** Primer sequences of KP-OL-CDA and PCR targeting the map gene of *Klebsiella pneumoniae*.

Target	Method	Primer	Sequence (5′ → 3′)
*Klebsiella pneumoniae rcs*A gene	CDA	KP-MF	ACAGTGTTTCTGCAAAACAGGAAATCGTTGAGG
		KP-MR	ACGTCGTGTTGCCCGAATAATGCCATTACTTTC
		KP-F2	GATTATGGATTTGTGCAGC
		KP-R2	GGTTTTGCGTAATGATCTG
		KP-LR	TCATGCACGAAACAGTCTTC
	PCR	KP-F	GTTGGGATTGACGGGAT
		KP-R	AAACTGAACTGGCGGGA

### 2.4 CDA reaction

The standard CDA reactions were performed according to the method previously reported by our research group. The CDA reaction mixture consisted of 12.5 μL of 2 × isothermal amplification buffer [including 40 mM Tris–HCl, 20 mM KCl, 20 mM (NH_4_)_2_SO_4_, 16 mM MgSO_4_, and 0.2% Triton X-100], 1 μL MF (40 μM), 1 μL MR (40 μM), 1 μL *Bst* 2.0 WarmStart DNA polymerase, 1 μL for each EvaGreen or/and hydroxy naphthol blue (HNB), a proper number of nucleic acid templates, and RNase-free water up to 25 μL. Finally, 30 μL of paraffin oil was added to avoid possible cross-contamination from the aerosol of CDA product leakage. The amplification reaction was carried out under isothermal conditions (60 ~ 65°C) for 60 min and then terminated at 85°C for 10 min. The CFX96 Real-Time PCR detection system (California, USA: Bio-Rad) and Q3 Real-Time PCR instrument (California, USA: Applied Biosystems) were used for real-time fluorescence monitoring of CDA reactions and final melting curve analysis of the CDA products. Direct visual observation was adopted to detect CDA reaction results by adding the indicator HNB to the mixture. The post-CDA amplification mixture of colors shifted from purple to sky blue, indicating the production of insoluble magnesium pyrophosphate during the nucleic acid synthesis. In addition, the CDA products were analyzed by 1% agarose gel electrophoresis and stained with GelRed, and images were visualized using an ultraviolet (UV) transilluminator. For O-CDA reactions, other outer primers (0.2 μM) were added to the former CDA mixtures, and for reactions, other outer primers (0.2 μM) and loop primers (0.8 μM) were added to the former CDA mixtures. The negative control (NC) contained non-target nucleic acid samples or nuclease-free water.

### 2.5 Optimal amplification temperature of the established KP-CDA assay

To select the optimal temperature for the *K. pneumonia* CDA assay, identical reactions were performed at various temperatures (60, 61, 62, 63, 64, and 65°C, separately). The real-time fluorescence curves of each *K. pneumoniae* CDA reaction were recorded, and distilled water instead of target DNA samples were used as NCs to explore the merits of non-specific amplification of the developed assay. The temperature to carry on CDA amplification of the extracted *K. pneumoniae* DNA with a minimum cycle threshold (Ct) was chosen as the optimal temperature for the assay. The optimal temperature determination tests were repeated four times.

### 2.6 Determination of the sensitivity and specificity of the KP-CDA assay

The sensitivity of the developed CDA method was explored using the gradient-diluted DNA samples. The extracted *K. pneumonia* genomic DNA from clinical samples ranged from 1.2 × 10^−5^ ng/μL to 12 ng/μL with sterile water. Real-time fluorescence curves of 14 different DNA concentrations were assessed to determine the detection limit of the developed CDA method. NCs were included in every batch, consisting of double-sterilized water or non-target DNA samples to ensure there was no occurrence of non-specific amplification. In addition, the amplification products were further analyzed through gel electrophoresis.

To evaluate the specificity of the CDA method, 30 clinical strains of *K. pneumoniae* DNA and 14 non-*K. pneumoniae* strains (*Staphylococcus aureus, Shigella sonnei, Vibrio parahaemolyticus, Escherichia coli, Candida glabrata, Candida tropicalis, Candida parapsilosis, Candida albicans, Streptococcus agalactiae, Rickettsia raoultii, Listeria monocytogenes, Pseudomonas aeruginosa, Klebsiella oxytoca*, and *Klebsiella aerogenes*, listed in Table 1) were tested by the developed CDA method. The DNA of all strains was extracted using DNA purification kits. The CDA experimental results were obtained through real-time amplification curves, followed by a melting curve analysis of the CDA products and end-point colorimetric-based evaluations.

### 2.7 Real-time quantitative PCR assays

The extracted *K. pneumoniae* samples were amplified by real-time quantitative PCR (forward primer: CTACTATTATTATCGCCCGC and reverse primer: CCGCCAGTTTGTTTCAG) (Table 3). A 50-μL reaction volume was used for real-time quantitative PCR (qPCR), including the following components: 25 μL of 2 × Taq PCR Mix (Sangon, Shanghai, China), 1 μL for each primer (40 μM), 2 μL for the target, 1× EvaGreen (1 μL), and final nuclease-free water were used to adjust the volume to 50 μL. The PCR cycling conditions are denaturation at 95°C for 2.5 min, annealing at 55°C for 30 s, and extension at 72°C for 20 s, for a total of 35 cycles. Final concentration to achieve real-time fluorescence monitoring in the CFX96 Real-Time PCR detection system (Bio-Rad) and Q3 Real-Time PCR instrument (Applied Biosystems). The PCR conditions of the commercial *Klebsiella Pneumoniae* Real-Time PCR Kit from Shanghai Liferiver are maintaining at 37°C for 2 min, denaturation at 93°C for 15 s, and annealing and extension at 60°C for 60 s, for a total of 40 cycles. Moreover, identical DNA template dilutions were used to compare the sensitivity of the CDA assay, the PCR traditional assay, and the commercial PCR assay.

## 3 Results

### 3.1 Designing and screening of CDA primers for *K. pneumoniae* detection

**Figure 2:**
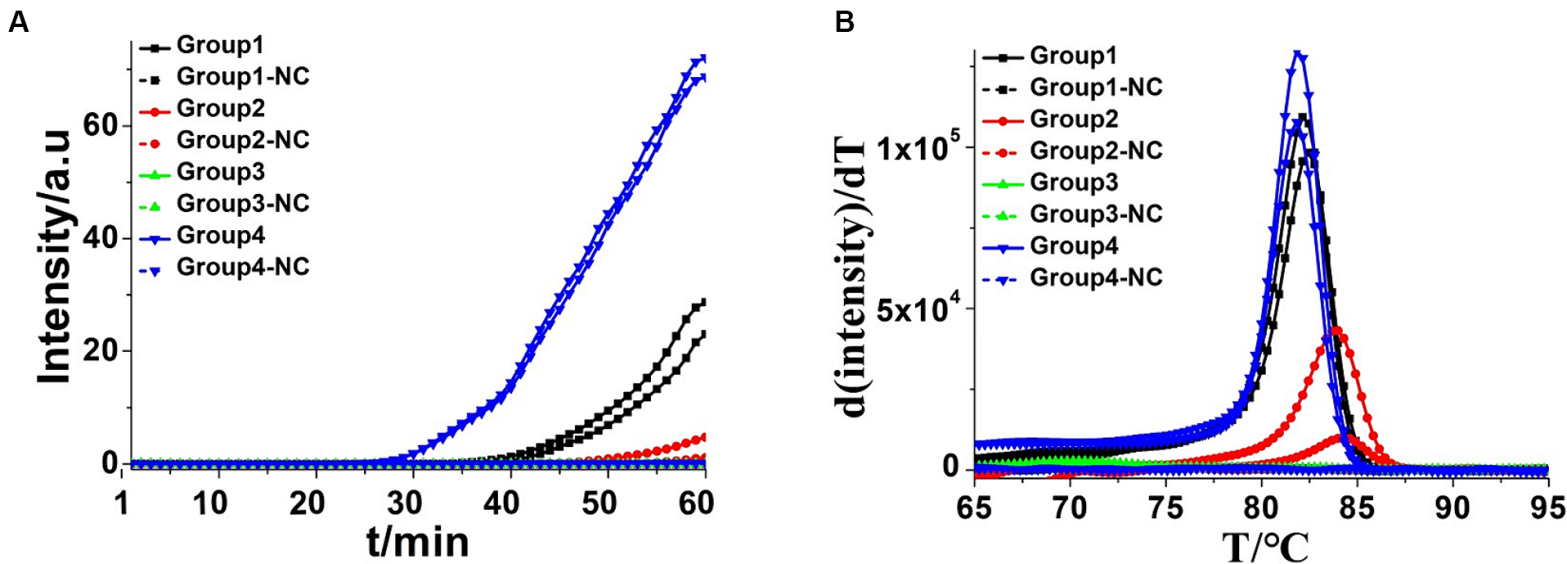
Amplifications of extracted *Klebsiella pneumoniae* DNA by CDA monitored by real-time PCR carried out at 60°C for 60 min. **(A)** Real-time CDA of different groups for *Klebsiella pneumoniae* DNA, Group 1 contained the primers of KP-MF-1 and KP-MR-1, and other groups contained the primers in order (in each set two positive reactions and two negative reactions). **(B)** Melting curve analysis of CDA products by real-time PCR. NC, negative control reaction without the targeted DNA.

Three batches of four pairs of CDA primers targeted at the *K. pneumoniae rcs*A gene were designed and selected to get the optimum primer set. The group of CDA reactions with a minimum cycle threshold determined by a real-time fluorescence monitoring curve was preferred for high amplification efficiency. As shown in Figure 2, the amplification curve of the CDA method revealed that the fourth group of primers could amplify the target gene in the shortest time and were selected as the optimal primers for *K. pneumoniae* with the CDA assay (Table 3). The threshold detection time of 1.2 ng/μL of the extracted *K. pneumoniae* DNA by the determined primers was 29 min, the melting temperature of the amplified product was 82.0°C, and no non-specific amplifications were observed in NCs. Both real-time amplification curves and melting curves showed that the developed CDA method for *K. pneumoniae* detection exhibited good repeatability and stability. In addition, a developed CDA assay combined with HNB was applied for simple visual detection of *K. pneumoniae.* In the HNB-based reaction mixture, the positive reaction is indicated by a color change from purple to light blue, whereas the negative reaction retains the purple color.

### 3.2 Optimization of the CDA assay for *K. pneumoniae*

**Figure 3:**
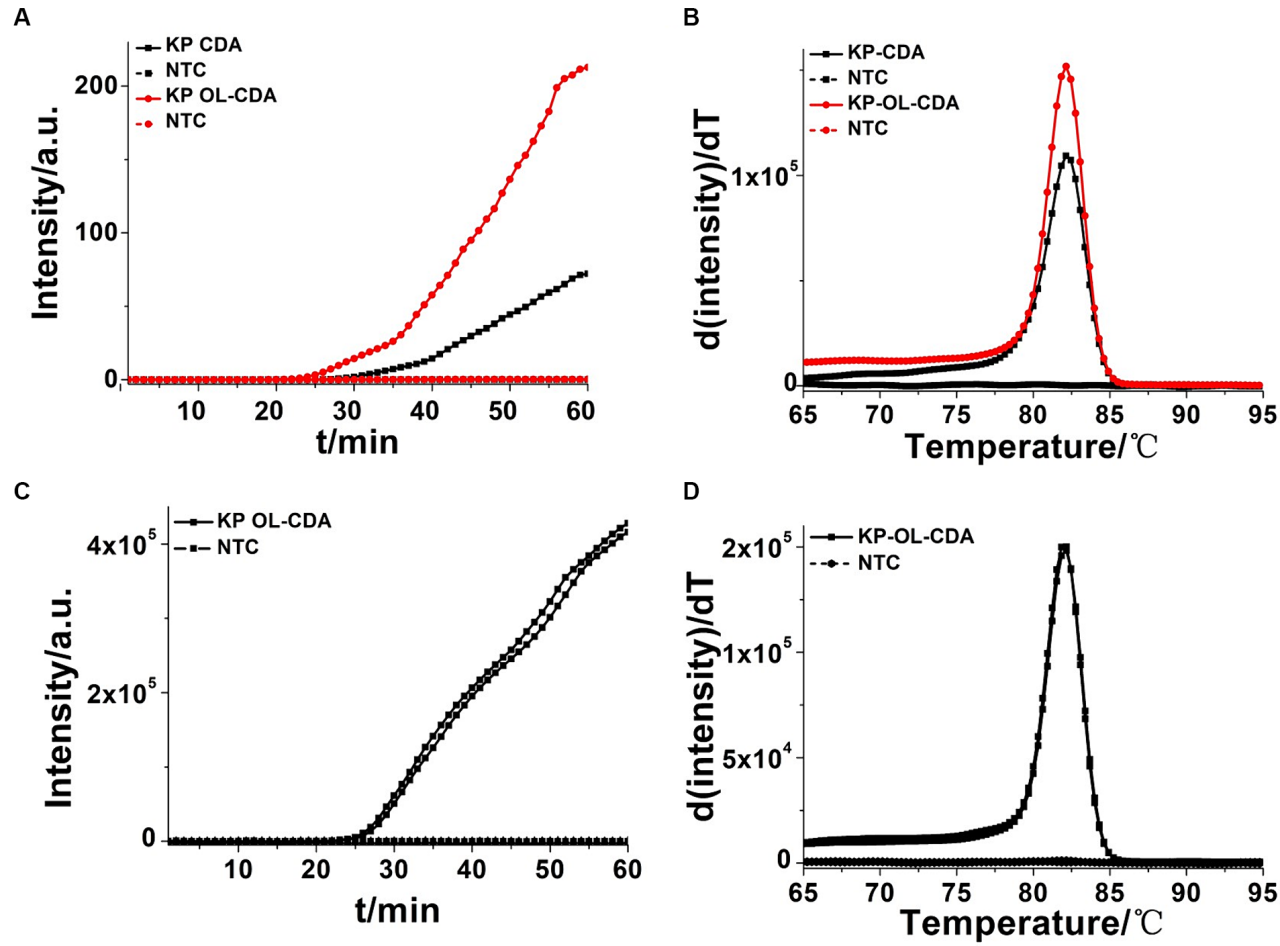
Amplifications of extracted *Klebsiella pneumoniae* DNA by KP-CDA and KP-OL-CDA monitored by real-time PCR. **(A)** Amplification plot of real-time KP-CDA and KP-OL-CDA for extracted DNA. **(B)** Melting curve analysis of KP-CDA and KP-OL-CDA products. **(C)** Repeatability analysis of the KP-OL-CDA monitored by real-time PCR at 60°C. **(D)** Repeatability analysis of melting curve analysis of KP-OL-CDA products. NTC, negative control reaction without the targeted DNA.

**Figure 4:**
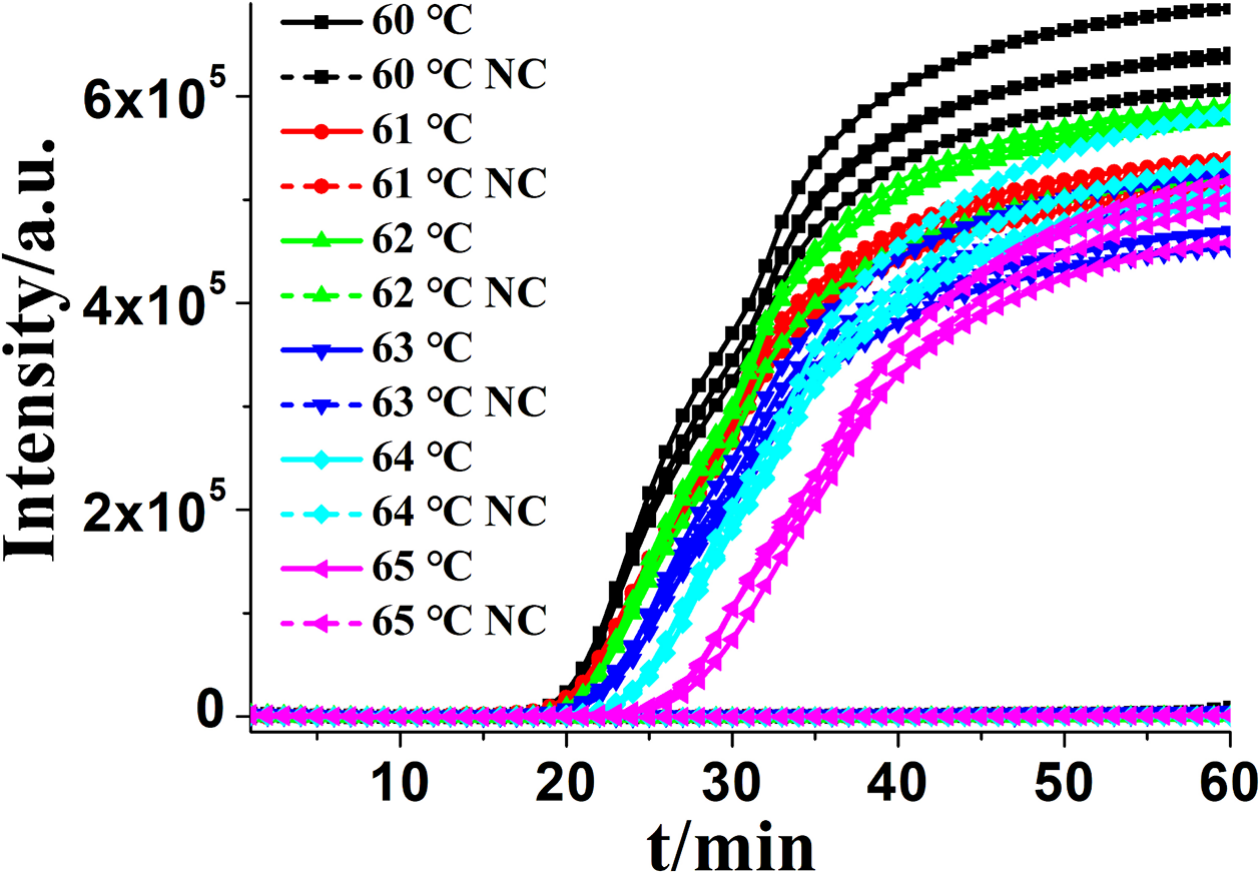
Amplification plot of real-time KP-OL-CDA for *Klebsiella pneumoniae* at different temperatures. Each set exhibited four positive reactions. NC, negative control, negative control reaction without the targeted DNA.

Accelerating primers (outer primers and loop primers) were added to the CDA reaction mixtures to achieve more efficient *K. pneumoniae* detection approaches, termed as OL-CDA. As shown in Figure 3A, the threshold time of detecting *K. pneumoniae* (1.2 ng/μL) was shortened by 4 min using the OL-CDA method, indicating that the reaction efficiency of the CDA method was improved by adding extra accelerating primers. After the identification of accelerated primers, the optimal temperature for an efficient *K. pneumoniae* OL-CDA assay was determined by incubating for 60 min at temperatures of 60, 61, 62, 63, 64, and 65°C, with four replicates at each temperature, respectively. The optimal reaction temperature for the developed OL-CDA method was set at 60°C to ensure perfect detection (Figure 4).

To further assess the repeatability of the developed assay, 12 ng/μL of the extracted *K. pneumoniae* DNA was amplified in eight positive and negative replicates independently through OL-CDA. Both the real-time amplification curve (Figure 3C) and melting curve (Figure 3D) indicated good repeatability of the OL-CDA method. In addition, the melting curves, as shown in Figures 3B,D, were completely identical, indicating that the accelerated primers would help to finish the amplification reaction in a shorter time without the introduction of non-specific amplification.

### 3.3 Sensitivity and specificity assay for CDA and PCR of *K. pneumoniae*

**Figure 5:**
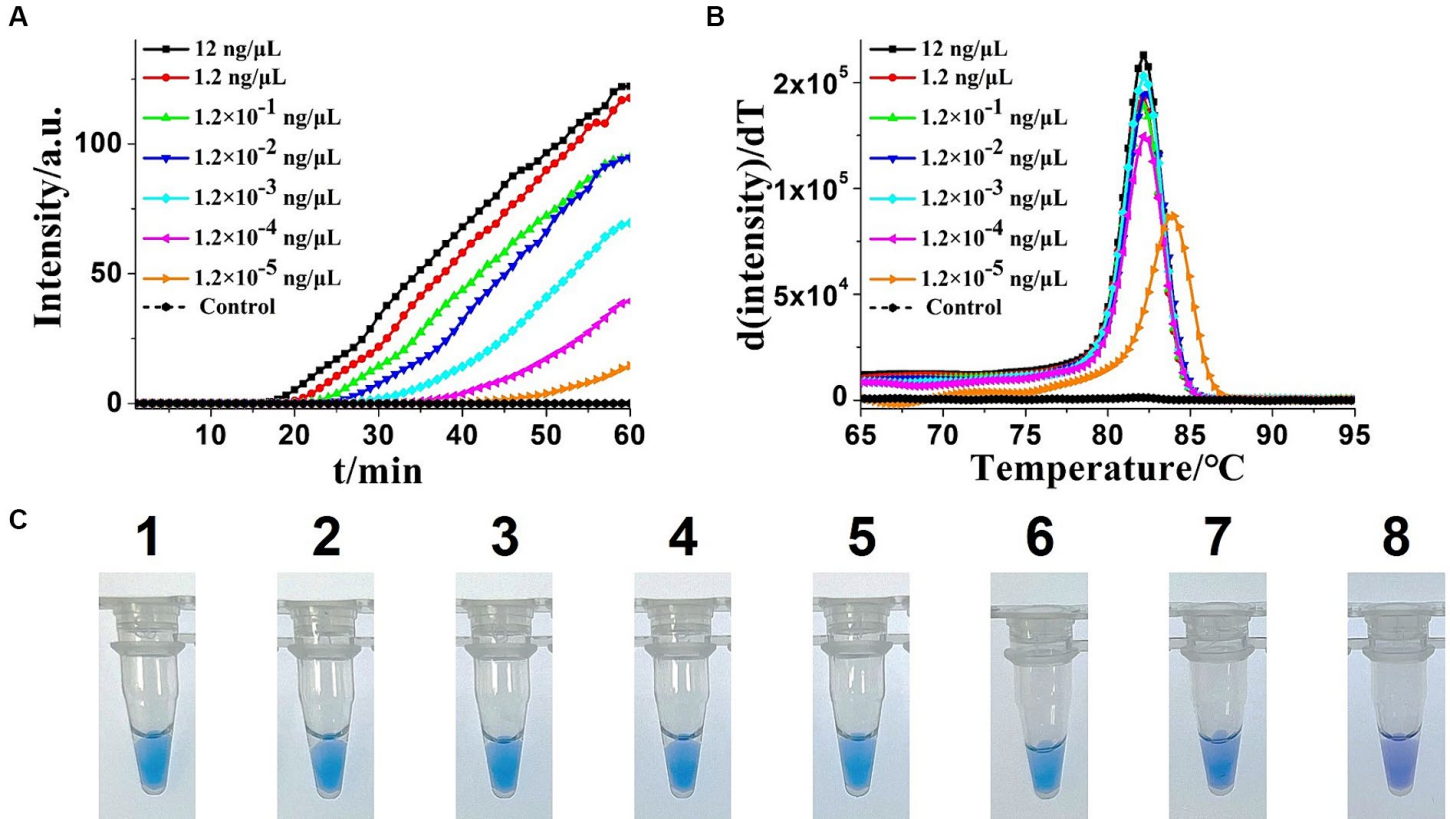
Sensitivity analysis of the extracted *Klebsiella pneumoniae* DNA KP-OL-CDA method by real-time and visual approaches. **(A)** KP-OL-CDA amplification is monitored by real-time PCR every 1 min at different concentrations of DNA. The reaction was performed at 60°C for 60 min. **(B)** Melting curve analysis of KP-OL-CDA products at different concentrations of DNA. **(C)** Sensitivity analysis of *Klebsiella pneumoniae* detection by visual detection of KP-OL-CDA. The numbers 1–8 presented the DNA concentrations were as follows: 12, 1.2, 1.2 × 10^−1^, 1.2 × 10^−2^, 1.2 × 10^−3^, 1.2 × 10^−4^, 1.2 × 10^−5^ ng/μL. NC, negative control reaction without the targeted DNA.

**Figure 6:**
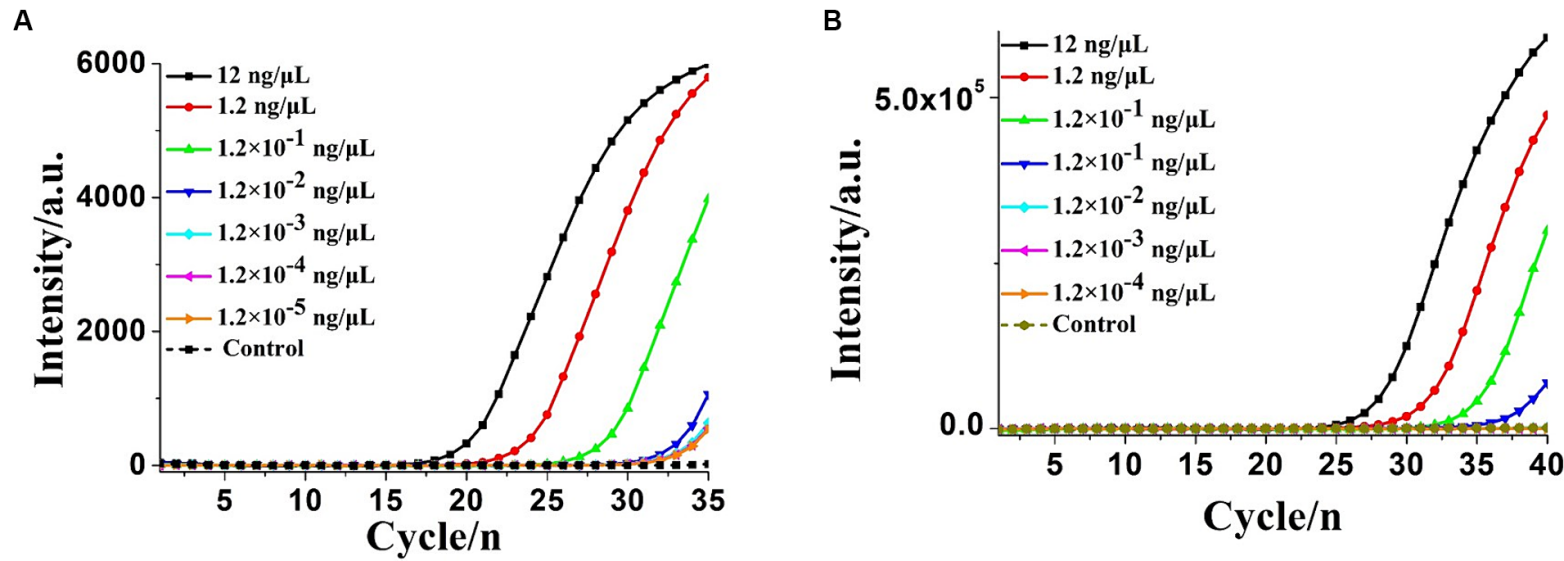
Real-time PCR assay for extracted *Klebsiella pneumoniae* DNA detection. **(A)** Real-time amplification plot by the PCR method developed in this study. **(B)** Real-time amplification plot using the commercial real-time quantitative PCR (qPCR) assay. NC, negative control reaction without the targeted DNA.

To determine the sensitivity of the OL-CDA assay, the DNA concentration of 10-fold diluted extracted *K. pneumoniae* DNA ranging from 12 to 1.2 × 10^−5^ ng/μL was prepared. As shown in Figures 5A,C, the amplification curve and the color change of the reaction products indicated that the limit of the OL-CDA method for *K. pneumoniae* was 1.2 × 10^−5^ ng/μL, which corresponds to approximately one copy of the target gene in the reaction system (Zong et al., 2012). In addition, the reproducibility of detection limit evaluation showed that the established OL-CDA method could amplify 1.2 × 10^−4^ ng/μL of *K. pneumoniae*-extracted genomic DNA. This repeatability was confirmed through real-time amplification (Figure 5A) and end-point visualization (Figure 5C) analyses. In comparison, the detection limit of the real-time qPCR assay using the specific primers F1 and R1 was 1.2 × 10^−3^ ng/μL of the extracted DNA (Figure 6A). The detection limit of the commercial real-time qPCR assay was 1.2 × 10^−3^ ng/μL of the extracted DNA (Figure 6B). Thus, the developed CDA assay was 100-fold more sensitive than the traditional PCR and 1,000-fold more sensitive than the commercial real-time qPCR assay. In addition, the melting curve analysis of *K. pneumoniae* using the real-time fluorescence OL-CDA method showed that the Tm values of 1.2 × 10^−5^ ng/μL of the extracted DNA were slightly different from other concentrations, which might be affected by a high concentration of non-targeted DNA sequences (Figure 5B).

**Figure 7:**
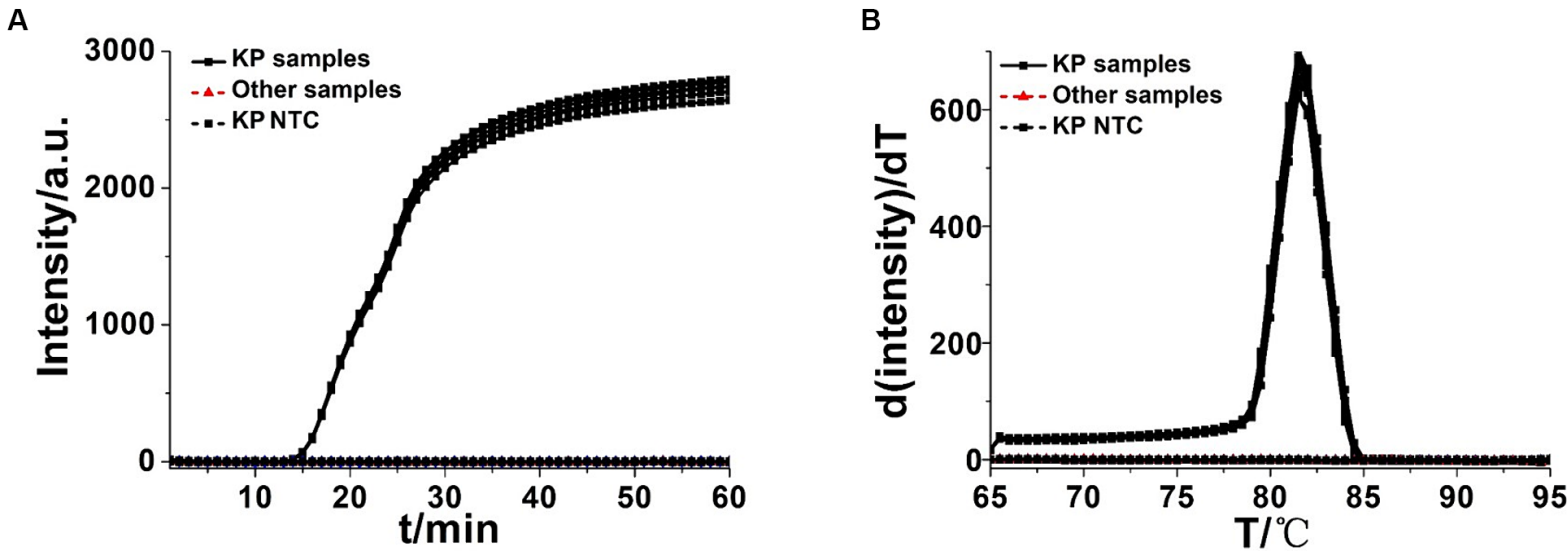
Real-time KP-OL-CDA assay. Real-time KP-OL-CDA reactions were carried out at 60°C for 60 min. **(A)** Real-time KP-OL-CDA for extracted *Klebsiella pneumoniae* DNA positive controls (KP samples), clinical *Klebsiella pneumoniae* strains, other samples (*Staphylococcus aureus, Shigella sonnei, Vibrio parahaemolyticus, Escherichia coli, Candida glabrata, Candida tropicalis, Candida parapsilosis, Candida albicans, Streptococcus agalactiae, Rickettsia raoultii, Listeria monocytogenes, and Pseudomonas aeruginosa*), and negative control reaction without the targeted DNA (NTC). **(B)** Melting curve analysis of the KP-OL-CDA products by real-time PCR. NTC, negative control reaction without the targeted DNA.

**Figure 8:**
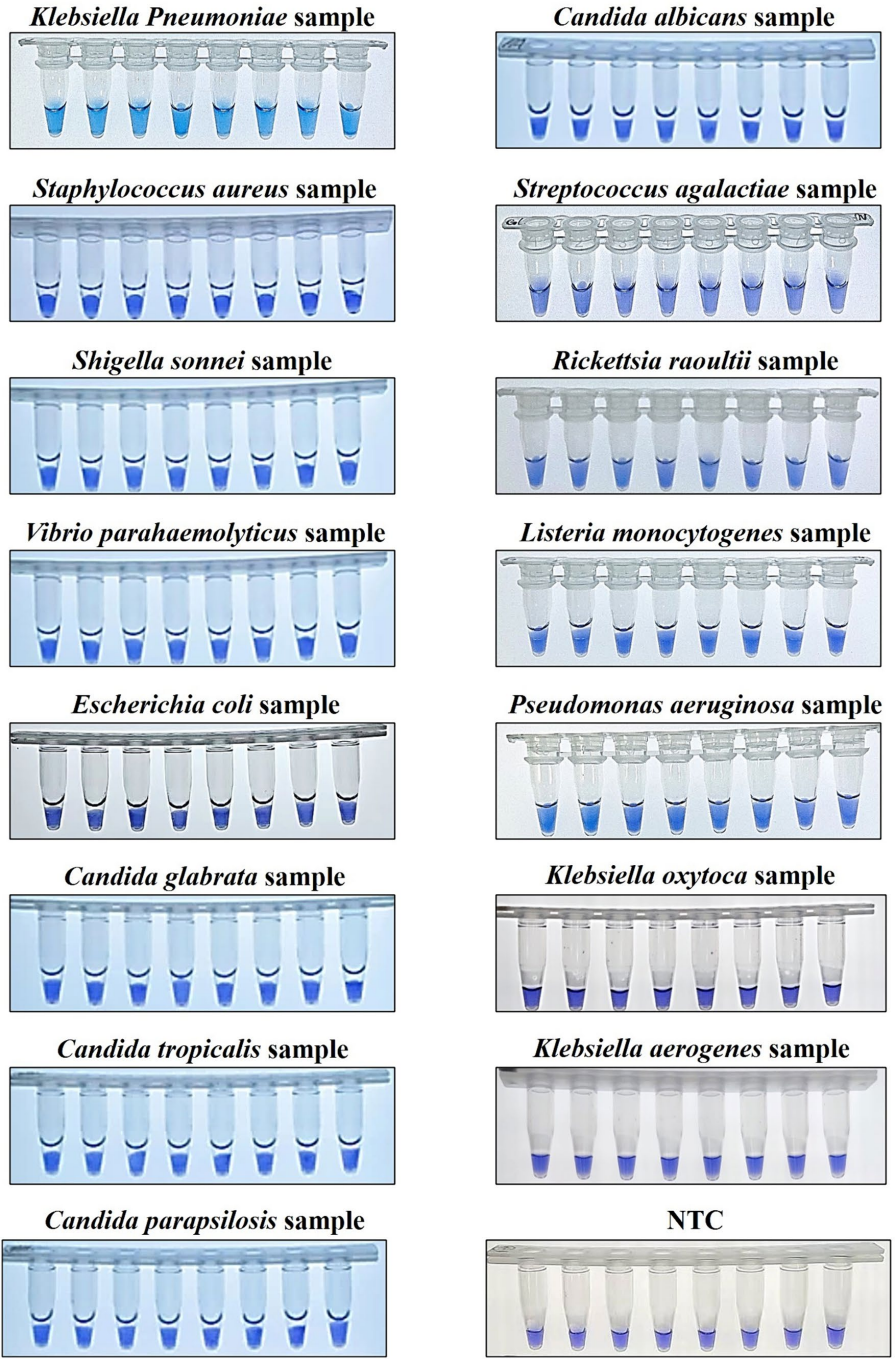
Colorimetric KP-OL-CDA assay using HNB. Line 1, positive controls (12 ng/μL of templates); Line 2, DNA samples extracted from *Staphylococcus aureus*; Line 3, DNA samples extracted from *Shigella sonnei*; Line 4, DNA samples extracted from *Vibrio parahaemolyticus*; Line 5, DNA samples extracted from *Escherichia coli*; Line 6, DNA samples extracted from *Candida glabrata*; Line 7, DNA samples extracted from *Candida tropicalis*; Line 8, DNA samples extracted from *Candida parapsilosis*; Line 9, DNA samples extracted from *Candida albicans*; Line 10, DNA samples extracted from *Streptococcus agalactiae*; Line 11, DNA samples extracted from *Rickettsia raoultii*; Line 12, DNA samples extracted from *Listeria monocytogenes.* Line 13, DNA samples extracted from *Pseudomonas aeruginosa*; Line 14, DNA samples extracted from *Klebsiella oxytoca*; Line 15, DNA samples extracted from *Klebsiella aerogenes*; Line 16, No template controls (RNase-free water). Due to space limitations, other results were not displayed. HNB, hydroxy naphthol blue.

To assess the specificity of the OL-CDA assay for detecting *K. pneumoniae* by evaluating its reactivity with non-*K. pneumoniae* strains, the assay specifically amplified their DNA sequences from *K. pneumoniae* without cross-reaction with other non-*K. pneumoniae* strains (Figure 7A). The melting curve analysis was consistent with the former results (Figures 3B,D, 7B), and there was no difference between the positive control and different DNA samples, which further illustrated the good reproducibility of the developed *K. pneumoniae* CDA detection method. In addition, 30 clinical strains were adopted to evaluate the repeatability, practicability, and reliability of the developed OL-CDA method. Furthermore, the mixture of non-*K. pneumoniae* genomic DNAs and tissue sample DNAs was set as NCs to evaluate the solidity of the established method. The results showed that the newly established OL-CDA method can effectively detect *K. pneumoniae* in different non-*K. pneumoniae* strains and various NCs by both real-time fluorescence and visual end-point approaches (Figures 7, 8). The results showed that the developed CDA diagnostic assays for *K. pneumoniae* detection had high reaction efficiency, robustness, sensitivity, and specificity (both 100%) for a total of 520 batches of tests (Table 4).

**Table 4:** Determination of sensitivity and specificity of the KP-CDA assay for *Klebsiella pneumoniae*.

Species	Sample numbers		Sensitivity	Specificity	Accuracy
	KP-CDA	Defined	95% CI^*^	95% CI^*^	95% CI^*^
*Klebsiella pneumoniae*	296	296	1.0 (98.8–100.0)	1.0 (98.4–100.0)	1.0 (99.3–100.0)
*Staphylococcus aureus*	0	16			
*Shigella sonnei*	0	16			
*Vibrio parahaemolyticus*	0	16			
*Escherichia coli*	0	16			
*Candida glabrata*	0	16			
*Candida tropicalis*	0	16			
*Candida parapsilosis*	0	16			
*Candida albicans*	0	16			
*Streptococcus agalactiae*	0	16			
*Rickettsia raoultii*	0	16			
*Listeria monocytogenes*	0	16			
*Pseudomonas aeruginosa*	0	16			
*Klebsiella oxytoca*	0	16			
*Klebsiella aerogenes*	0	16			
Total	296	520			

^*^CI, confidence interval. Statistical analysis was carried out by an online program called Diagnostic Test Evaluation Calculator (https://www.medcalc.org/calc/diagnostic_test.php).

## 4 Discussion

*Klebsiella pneumoniae* is a well-recognized nosocomial infected pathogen and a member of the ESKAPE group of antimicrobial-resistant pathogens (*Enterococcus faecium*, *Staphylococcus aureus*, *Klebsiella pneumoniae*, *Acinetobacter baumannii*, *Pseudomonas aeruginosa*, and *Enterobacter* species). These pathogens seriously threaten human health, and the drug resistance of these pathogens presents a significant therapeutic challenge (Bialek-Davenet et al., 2014; Mulani et al., 2019; De Oliveira et al., 2020). Therefore, early and rapid diagnosis of *K. pneumoniae* is beneficial for guiding clinical treatment, medication, and effective control of infections.

In recent years, reports regarding an increasing number of rapid detection methods for *K. pneumoniae* have been optimized constantly based on the LAMP assay (Griffioen et al., 2020; Rivoarilala et al., 2021). In the research field of isothermal amplification, LAMP-based diagnosis methods have exhibited the advantages of being rapid, simple, and accurate. Moreover, LAMP reactions were established by selecting 6–8 regions of the target gene, making them more specific and sensitive than the traditional PCR method (Solanki et al., 2013). However, the development of LAMP-based diagnosis methods was restricted by the complexity of designing primers and non-specific amplification (Poirier et al., 2021). In this study, we developed a simple CDA assay with high sensitivity and specificity, which would contribute to the rapid detection and on-site surveillance of *K. pneumoniae* infection in the clinical environment. In this study, we successfully established and optimized a CDA method for the rapid detection of *K. pneumoniae* by amplification of the specific *rcs*A gene (Su et al., 2018). In the CDA-based detection method, primers were designed and selected according to the CDA criteria, targeting the conserved *rcs*A gene of *K. pneumoniae*. The visual detection of *K. pneumoniae* through CDA also showed perfect repeatability.

Three important diagnostic indexes (detection limit, sensitivity, and specificity) of the developed *K. pneumoniae* CDA diagnostic assays were evaluated as common molecular diagnosis methods. The specificity test indicated that all 30 clinical *K. pneumoniae* strains were found to be positive and non-*K. pneumoniae* strains (*Staphylococcus aureus, Shigella sonnei, Vibrio parahaemolyticus, Escherichia coli, Candida glabrata, Candida tropicalis, Candida parapsilosis, Candida albicans, Streptococcus agalactiae, Rickettsia raoultii, Listeria monocytogenes, Pseudomonas aeruginosa, Klebsiella oxytoca*, and *Klebsiella aerogenes*) were found to be negative using the CDA assay. Furthermore, the detection limit of the *K. pneumoniae* CDA assay was determined to be 1.2 × 10^−5^ ng/μL of the extracted DNA sample, which corresponds to approximately one copy of the target gene in the reaction mixture. The detection limit of *K. pneumoniae* found using the CDA assay was 100–1,000 times lower than that of the corresponding PCR assay and the commercial PCR assay mentioned in this study.

Furthermore, the sensitivity and specificity in the detection of *K. pneumoniae* were both 100% from a total of 520 tests. Regarding the reported LAMP and recombinase-aided amplification (RAA)-based *K. pneumoniae* diagnostic assays, Schneider et al. (2019) evaluated that the LAMP method had a high rate of false-positive results due to the complicated primer design and amplification system. Hou et al. (2021) found that the accuracy of the RAA assay was 96.9%, with two false-positive results in a total of 64 batches evaluated. The developed *K. pneumoniae* CDA assays exhibited outstanding performance compared to the reported LAMP and RAA assays, with limited clinical sample evaluation in statistics. Overall, the developed assays may be suitable for primary hospitals with limited resources and for site inspections in disease control and prevention departments. Finally, to achieve simultaneous and multiplex detection of mixed infections, the CDA method coupled with lateral flow dipstick-based approaches would play a significant role (Mabrok et al., 2021).

In conclusion, a rapid, sensitive, specific, and convenient technology based on the CDA method for the detection of *K. pneumoniae* DNA was successfully established in this study. Considering the convenient operation of an HNB-based colorimetric detection system, a CDA assay for *K. pneumoniae* would be a potential measure for on-site diagnostic assays in underdeveloped regions.

## Data availability statement

The original contributions presented in the study are included in the article/supplementary material, further inquiries can be directed to the corresponding author.

## Ethics statement

The studies involving humans were approved by the Ethics Committee of Clinical Investigation in the First Affiliated Hospital of Ningbo University. The studies were conducted in accordance with the local legislation and institutional requirements. Written informed consent for participation in this study was provided by the participants' legal guardians/next of kin.

## Author contributions

YZ: Conceptualization, Investigation, Methodology, Resources, Writing – original draft, Writing – review & editing. XC: Investigation, Methodology, Writing – review & editing. GO: Investigation, Methodology, Writing – review & editing. JW: Writing – review & editing. YS: Writing – review & editing. YL: Writing – review & editing. PZ: Writing – review & editing. FG: Writing – review & editing. SY: Supervision, Writing – review & editing. RM: Investigation, Methodology, Project administration, Supervision, Writing – original draft, Writing – review & editing.

## Footnotes

^1^http://cels.uri.edu/gsc/cndna.html
